# An Ultrasonographic Study of the Superficial Radial Nerve in Healthy Subjects: Suggesting a Safe Zone for Wrist Extensor Compartment Injections

**DOI:** 10.3390/diagnostics16121788

**Published:** 2026-06-10

**Authors:** So Hyun Park, Jae Eun Chang, Joon Shik Yoon

**Affiliations:** Department of Physical Medicine and Rehabilitation, Korea University Guro Hospital, College of Medicine, Korea University, Seoul 08308, Republic of Korea; parksh0406g@gmail.com (S.H.P.); jenny1474@naver.com (J.E.C.)

**Keywords:** superficial radial nerve, De Quervain’s tenosynovitis, intersection syndrome, ultrasonography, safe zone, iatrogenic injury

## Abstract

**Background/Objectives:** The superficial radial nerve (SRN) is highly susceptible to iatrogenic injury during wrist injection procedures. This study aimed to identify the anatomical trajectory of the SRN using high-resolution ultrasonography and to establish a reliable “safe zone” for wrist extensor compartment injections. **Methods:** Fifty-eight forearms from 29 healthy volunteers (15 males, 14 females) were evaluated. Four anatomical levels were defined: the proximal and distal ends of the extensor compartment I-II intersection area (Levels A and B), and the proximal and distal points of SRN crossing over the first compartment (Levels C and D). Longitudinal distances from the radial styloid, horizontal distances and depths of the SRN were measured. Generalized Estimating Equations (GEEs) were used to analyze the relationship between total forearm length and the longitudinal position of each landmark. **Results:** Total forearm length was significantly associated with proximal landmarks, *L*a (*B* = 0.205, *p* < 0.001) and *L*c (*B* = 0.105, *p* < 0.001). Although *L*b also showed a significant association (*B* = 0.071, *p* = 0.019), its absolute variation was minimal. The most distal landmark *L*d (*B* = −0.023, *p* = 0.610) exhibited no significant relationship. For intersection syndrome, a safe injection corridor was identified between 24.1% and 12.7% of forearm length (Level A to C), where a proximal-to-distal and dorsal-to-volar needle direction is recommended, as the SRN lies volar at this level. For De Quervain’s tenosynovitis, a volar-to-dorsal needle direction at or distal to 0.8 cm from the radial styloid (Level D) minimizes nerve contact risk. **Conclusions:** This study suggests a differentiated, landmark-based approach for wrist injections: utilizing proportional ratios for proximal landmarks and fixed absolute distances for distal landmarks. This individualized guide is expected to enhance procedural safety and minimize the risk of iatrogenic SRN injury.

## 1. Introduction

The superficial radial nerve (SRN) is a purely sensory branch of the radial nerve that provides cutaneous innervation to the dorsal surface of the thumb, index, middle and radial half of the ring finger [1]. Due to its superficial location in the distal forearm, it is highly susceptible to external trauma and iatrogenic injury during clinical procedures [2].

The SRN branches from the radial nerve at the level of the proximal forearm, subsequently passing under the brachioradialis (BR) muscle. In the distal third of the forearm, the nerve penetrates the antebrachial fascia, emerging between the tendons of the BR and the extensor carpi radialis longus (ECRL). It then travels superficially over the abductor pollicis longus (APL) and extensor pollicis brevis (EPB) tendons—which constitute the first extensor compartment—before branching toward the hand [3,4,5].

De Quervain’s tenosynovitis is one of the most common overuse injuries of the wrist [6]. It is characterized by the stenosing inflammation of the first extensor compartment, where restricted gliding of the APL and EPB tendons triggers mechanical pain and impingement [7]. Intersection syndrome represents another overuse pathology of the wrist, which involves inflammation at the crossover point of the first (APL, EPB) and second (ECRL, ECRB) extensor compartments [8,9]. Although corticosteroid injections are frequently used to manage these pathologies [10,11,12], the close proximity of the SRN to these extensor compartments poses a significant risk of iatrogenic injury during blind injection procedures [13,14]. Such injuries to the SRN can lead to persistent paresthesia or painful neuroma formation, significantly complicating the patient’s recovery [15].

Several cadaveric and ultrasonographic studies have described the general anatomical course of the SRN in the forearm using fixed bony landmarks [3,4,5,14]. However, most studies reported distances as absolute values in centimeters, without accounting for individual variation in forearm length. As forearm length may vary according to sex and height, using only absolute distances limits the generalizability across diverse populations.

Moreover, while ultrasound-guided injections enhance accuracy and safety [16,17,18], real-time imaging technology is not always available in every clinical setting, particularly in primary care or emergency departments. In such environments, identifying reliable, palpable anatomical landmarks to predict the trajectory of the tendons and the SRN would be of great clinical value [19]. To date, however, to the best of our knowledge, research providing a standardized, landmark-based guide that accounts for individual physical proportions—particularly the relationship between the SRN and the extensor compartments in the context of injection procedures—remains limited.

The objective of this study is to assess the anatomical relationship between the SRN and adjacent structures using high-resolution ultrasonography and to suggest a “safe zone” for injection procedures in the wrist extensor compartments. We hypothesized that this safe zone can be defined as a proportional ratio relative to the total forearm length, thereby providing a customized anatomical guide that accounts for individual physical differences.

## 2. Materials and Methods

### 2.1. Subjects

This study was conducted from February 2026 to May 2026 on 58 forearms from 29 healthy volunteers (15 males and 14 females) aged over 19 years. Exclusion criteria included individuals with a history of cervical radiculopathy, peripheral neuropathy, or previous trauma or surgical interventions involving the upper extremity.

We recruited volunteers from the local community via public announcements placed at Korea University Guro Hospital. Every participant signed a written informed consent prior to any examination. This study was approved by the Institutional Review Board of the Korea University Guro Hospital (IRB No. 2020GR0360).

Demographic data including age, sex, body height and weight were collected before performing the ultrasonographic assessment. The total length of forearm (*L*) of each side was measured as the distance between the radial styloid process and lateral epicondyle of the humerus, which could be palpated on the skin surface.

### 2.2. Ultrasonographic Measurement

All ultrasonographic assessments were conducted by a single rehabilitation physician with over 10 years of clinical experience in musculoskeletal ultrasonography. A 3 to 12 MHz linear array transducer on a high-resolution real-time ultrasound system (RS80A; Samsung Medison, Seoul, Republic of Korea) was utilized.

Participants were assessed bilaterally while in a seated position. The forearm was laid on the examination table with the elbow flexed at 90 degrees, and the forearm semi-pronated. The probe was orientated perpendicular to the border of radius.

**Figure 1:**
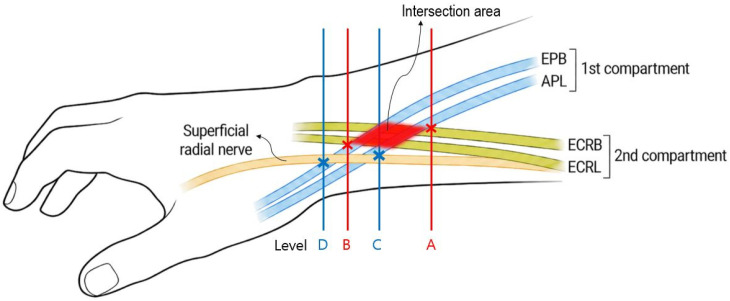
Schematic illustration of the anatomical relationship between the superficial radial nerve (SRN) and the wrist extensor compartments. The red ‘X’ marks denote the proximal (Level A) and distal (Level B) ends of the intersection area (red-shaded region), where the first (APL, EPB) and second (ECRL, ECRB) extensor compartments overlap. The blue ‘X’ marks indicate the proximal (Level C) and distal (Level D) points where the SRN crosses over the first extensor compartment. APL: abductor pollicis longus; EPB: extensor pollicis brevis; ECRL: extensor carpi radialis longus; and ECRB: extensor carpi radialis brevis.

The SRN was identified as it passed between BR and ECRL muscles [20,21]. The Doppler imaging was utilized to differentiate the nerve from adjacent vascular structures. The SRN was scanned in transverse plane and examined at four horizontal levels as illustrated in Figure 1. Level A and B were defined as the transverse planes passing through the proximal and distal ends of intersection area between the extensor compartment I and II, respectively. Specifically, Level A was designated as the proximal point where the APL tendon initiates to overlap the ECRB tendon. Level B was identified as the distal point where the EPB tendon terminates its crossing over the ECRL tendon. Level C and D represented the transverse planes passing through the proximal and distal crossing points of SRN over compartment I, respectively. Level C was defined as the most proximal contact point of ulnar border of SRN with radial border of APL, while Level D was identified as the most distal contact point between the radial border of SRN with the ulnar border of the EPB. In brief, Levels A and B define the longitudinal boundaries of the target zone for intersection syndrome, while Levels C and D indicate the points where the SRN crosses over the first extensor compartment.

**Figure 2:**
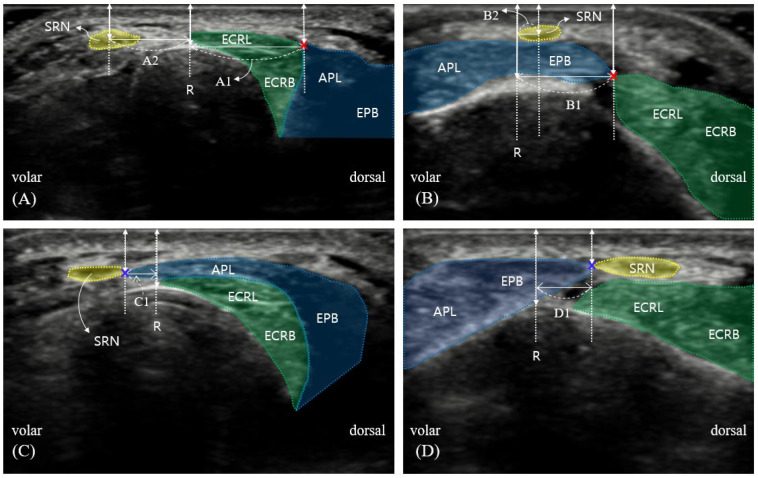
Representative ultrasonographic images of the wrist at the four designated levels (**A**–**D**): (**A**) Level A, (**B**) Level B, (**C**) Level C and (**D**) Level D. The red ‘X’ marks denote the intersection points at Levels A and B, while the blue ‘X’ marks indicate the SRN crossing points at Levels C and D. A1, A2, B1, B2, C1, and D1 indicate the horizontal distances from the superficial radial border to each respective landmark. Vertical white arrows indicate the depths from the skin surface to the SRN, intersection/crossing points, and the radius. R: radius; APL: abductor pollicis longus; EPB: extensor pollicis brevis; ECRL: extensor carpi radialis longus; and ECRB: extensor carpi radialis brevis.

Ultrasonographic measurements were performed at each level as demonstrated in Figure 2. At Level A and B, horizontal distances of the intersection point and SRN from the most superficial border of radius were measured (A1, A2, B1 and B2, respectively). The depths of the intersection points, SRN and the superficial border of radius were recorded from the skin surface. At Level C and D, the horizontal distances between the crossing point and the superficial border of radius were identified (C1 and D1, respectively). The depths of crossing point and superficial border of radius were measured from the skin surface as well. To minimize measurement error, the transducer was maintained strictly horizontal with minimal pressure applied to avoid tissue deformation. Upon identifying the transverse view for each level via ultrasonography, the corresponding locations were marked on the skin. After completion of all ultrasonographic evaluations, the longitudinal distances from the radial styloid process along the radial border to each skin mark were measured to determine *L*a, *L*b, *L*c, and *L*d. Proportions of these longitudinal distances relative to the total forearm length were calculated as *L*a/*L*, *L*b/*L*, *L*c/*L* and *L*d/*L*.

To evaluate intraobserver reliability, ultrasonographic measurements were repeated by the same examiner on a subset of 10 randomly selected participants (20 forearms) after a 1-month interval. Intraclass correlation coefficients (ICCs) were calculated using a two-way mixed-effects, absolute agreement, single-rater model [22].

### 2.3. Statistical Analysis

Statistical analyses were performed using SPSS, version 29.0 software (IBM Corp., Armonk, NY, USA). The normality of the data distribution was assessed using the Shapiro–Wilk test. The relationship between the total forearm length (*L*) and the longitudinal distances to each level was examined, and comparative analysis between male and female subgroups was performed. To account for the inter-limb dependence and potential within-subject clustering effects of evaluating bilateral forearms from the same individual, Generalized Estimating Equations (GEEs) were performed with the subject ID designated as the clustering variable. The GEE models were specified with a linear link function and an exchangeable working correlation structure. Statistical significance was defined as a *p*-value below 0.05. A post hoc power analysis was performed using G*Power software (version 3.1.9.7) to evaluate the adequacy of the sample size. The analysis was conducted within a linear multiple regression framework (random model) with a two-tailed significance level (α) of 0.05 and a total sample size of 58 forearms. The comprehensive raw data supporting the findings of this study are provided in File S1.

## 3. Results

Our study enrolled 29 healthy volunteers (15 males, 14 females), providing a total of 58 forearms for analysis. The baseline characteristics of the participants are presented in Table 1.

### 3.1. Intersection Area of Extensor Compartment I and II

The anatomical measurements regarding the intersection area are summarized in Table 2. Level A was located at a longitudinal distance of 6.2 ± 0.5 cm proximal from the radial styloid process, which corresponded to 24.1 ± 1.5% of the total forearm length (*L*). At this level, the proximal intersection point was 0.52 ± 0.13 cm dorsal to the superficial border of radius, whereas SRN was positioned 0.71 [0.65, 0.84] cm volar to the radius. Level B was identified at 1.2 ± 0.4 cm proximal from the styloid process (4.7 ± 1.6% of *L*). At this level, the distal intersection point was 0.32 ± 0.34 cm dorsal to the superficial border of the radius, while SRN was 0.08 [0.00, 0.17] cm dorsal to the radius. The depth of SRN was 0.50 ± 0.13 cm at level A and 0.25 [0.21, 0.30] cm at level B.

### 3.2. Crossing Points of SRN over Compartment I

**Figure 3:**
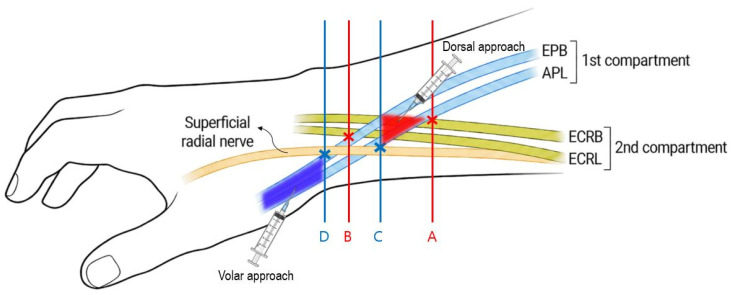
Optimal safe zones and needle approach strategies for injections into the first and second wrist extensor compartments. A–D represent the four anatomical levels as defined in Figure 1. The red-shaded region (between Levels A and C) indicates the safe zone for treating intersection syndrome. The blue-shaded region (distal to Level D) denotes the safe zone for De Quervain’s tenosynovitis. APL: abductor pollicis longus; EPB: extensor pollicis brevis; ECRL: extensor carpi radialis longus; and ECRB: extensor carpi radialis brevis.

The anatomical measurements of the crossing points of SRN over compartment I are presented in Table 3. Level C was measured at 3.2 ± 0.3 cm proximal from the styloid process (12.7 ± 1.2% of *L*), and Level D was located at 0.8 ± 0.4 cm (3.2 ± 1.8% of *L*). At level C, the proximal crossing point of SRN over APL was 0.45 ± 0.15 cm volar to the radius. At level D, the distal end of crossing was 0.25 [0.14, 0.36] cm dorsal to the radial border. In all cases, the SRN remained superficial to the first extensor compartment, following a diagonal course from volar to dorsal as it descended from proximal to distal. Based on these landmark-based measurements, Figure 3 illustrates the “safe zone” for injections into the first and second wrist extensor compartments.

The intraobserver reliability analysis demonstrated good-to-excellent reproducibility across all parameters (Supplementary Table S1). The ICC values for the longitudinal distances (*L*a, *L*b, *L*c, and *L*d) ranged from 0.894 to 0.921 (all *p* < 0.001), while the ICC values for the horizontal distances and depth measurements across all levels ranged from 0.755 to 0.969 (all *p* < 0.001).

### 3.3. Linear Relationships Between Forearm Length and Anatomical Landmarks

The GEE regression analysis was performed to evaluate the relationships between the total forearm length (*L*) and the longitudinal distances of each level (*L*a, *L*b, *L*c and *L*d) (Table 4). The total forearm length exhibited significant positive linear relationships with the proximal landmarks: *L*a (*B* = 0.205, *p* < 0.001) and *L*c (*B* = 0.105, *p* < 0.001), indicating that these landmarks shift distally as the forearm length increases. Additionally, *L*b demonstrated a minor but statistically significant positive relationship with forearm length (*B* = 0.071, *p* = 0.019). In contrast, the most distal landmark *L*d showed no statistically significant relationship with the total forearm length (*B* = −0.023, *p* = 0.610).

The post hoc power analysis confirmed that the sample size provided adequate statistical power to evaluate the primary hypotheses. Based on the observed effect sizes, the statistical power (1 − β) for the primary landmarks was 99.9% for *L*a (*f*^2^ = 0.608) and 98.2% for *L*c (*f*^2^ = 0.295). Both values exceed the conventional statistical power threshold of 80%, indicating that the sample size of 58 forearms was sufficient to minimize the risk of Type II errors.

### 3.4. Sex-Specific Comparisons of Anatomical Measurements

Anatomical measurements were compared between male and female participants to evaluate the influence of sex on forearm structures. Regarding the absolute longitudinal distances, males exhibited significantly longer measurements for *L*a (*B* = 0.590, *p* < 0.001), *L*b (*B* = 0.227, *p* = 0.046), and *L*c (*B* = 0.384, *p* < 0.001) compared to females. This trend is consistent with the significantly longer total forearm length observed in males (26.6 ± 1.2 cm vs. 24.4 ± 1.0 cm, *B* = 2.127, *p* < 0.001). However, no statistically significant differences were observed between sexes for the proportional ratios of *L*a/*L*, *L*b/*L*, *L*c/*L*, and *L*d/*L* (all *p* > 0.05). In terms of horizontal distances, no significant differences were found between men and women at all levels (*p* > 0.05). Regarding depth measurements, males exhibited statistically significant greater depths for a few specific anatomical structures: SRN at Level B (*B* = 0.046, *p* = 0.016), SRN crossing point at Level C (*B* = 0.040, *p* = 0.020), and superficial border of radius at Level D (*B* = 0.070, *p* = 0.011).

## 4. Discussion

This study quantified the anatomical relationship between the SRN and the wrist extensor compartments using high-resolution ultrasonography, demonstrating that the SRN consistently traverses compartment I in a volar-to-dorsal diagonal trajectory as it courses distally. The intersection area of the first and second extensor compartments was identified spanning from 24.1% to 4.7% of total forearm length proximal to the radial styloid (Level A to B). The SRN crossed over the first extensor compartment between approximately 12.7% (Level C) and 3.2% (Level D) of forearm length proximal from the radial styloid.

Previous cadaveric studies have described the course of the SRN using fixed bony landmarks. Samarakoon et al. reported that the SRN emerged from beneath the brachioradialis at an average distance of 8.54 cm proximal to the radial styloid process [3]. Similarly, Beldner et al. demonstrated that the SRN penetrated the antebrachial fascia at a mean of 9.3 cm in males and 8.3 cm in females proximal to the radial styloid [4]. This point of emergence was approximately 1.8 cm proximal to the intersection where the APL and EPB cross the ECRL and ECRB. Kim et al. demonstrated by ultrasonography that the SRN becomes subcutaneous at a mean of 10.31 cm from the radial styloid process [5]. However, none of these studies corrected for forearm length or examined the SRN trajectory in relation to the extensor compartments in the context of injection procedures.

Cheong et al. demonstrated in a cadaveric study that the lateral branch of the SRN overlapped with the APL tendon in up to 59% of specimens at the radial styloid level, confirming the anatomical vulnerability of the SRN during procedures near the first extensor compartment [14]. However, they measured distances only at two fixed points—the radial styloid and 1 cm proximal to it. Thus, their study did not investigate the precise longitudinal extent over which the SRN begins and terminates its crossing over the extensor compartment. To our knowledge, the present study is the first to quantify the SRN trajectory relative to both the first and second extensor compartments at the same time using in vivo ultrasonography. Compared to cadaveric studies, our ultrasonographic approach reflects living anatomy, accounting for the superficial displacement of the nerve under skin tension.

The use of proportional distances allows clinicians to apply these findings to patients with various statures. In our study, the total forearm length was significantly associated with *L*a (*B* = 0.205, *p* < 0.001) and *L*c (*B* = 0.105, *p* < 0.001). These results confirm that the proximal boundaries of the intersection area and the SRN crossing zone vary linearly in proportion to overall forearm size. In contrast, while *L*b showed a weak statistical relationship (*B* = 0.071, *p* = 0.019), its absolute change per centimeter of forearm length was minimal. Furthermore, *L*d exhibited no statistical relationship with forearm length (*B* = −0.023, *p* = 0.610). These findings suggest that the distal boundaries of these zones are relatively fixed in absolute position near the radial styloid. Collectively, these findings support the use of proportional ratios for the proximal landmarks (*L*a, *L*c), whereas distal landmarks (*L*b, *L*d) are better defined by their absolute distance from the radial styloid.

Anatomically, this regional divergence reflects upper extremity architectural scaling, where proximal landmarks are more influenced by muscle-tendon transitions that naturally adapt to overall musculoskeletal stature and forearm length. Conversely, distal landmarks are bound by rigid, osteofibrous structures, such as the extensor retinaculum and the radial styloid process, which maintain absolute locations regardless of overall body size. This localized stability is consistently observed in a recent cadaveric study reporting that the distance from the radial styloid process to the distal border of the extensor retinaculum over the first extensor compartment is narrowly limited to an average of 7.6 ± 1.8 mm [23].

The primary goal of this study was to provide an anatomical guideline to minimize SRN injury during wrist extensor compartment injections. Our results suggest two distinct safe zones based on the clinical indication.

For intersection syndrome, the injection target is the crossover area of the first and second extensor compartments, which spans from Level A to Level B (24.1% of forearm length to 4.7% proximal to the radial styloid). Although the entire intersection area is a potential injection target, the SRN begins to cross compartment I at Level C (12.7% of *L*), narrowing the safety margin in the distal portion of this zone. Therefore, the anatomical window between Level A and Level C—approximately 24.1% to 12.7% of forearm length proximal to the styloid—represents the relatively safe injection corridor for intersection syndrome. At Level A, the SRN is positioned significantly volar to the radial border (−0.71 cm), while the intersection point lies dorsal (+0.52 cm), creating a favorable separation between the nerve and the target. As the needle is directed distally, the SRN progressively migrates to a more dorsal and superficial position, with its depth decreasing from 0.5 cm at Level A to 0.33 cm at Level C. Accordingly, a proximal-to-distal with dorsal-to-volar needle direction initiated near Level A is recommended to maximize the distance from the SRN and improve procedural safety.

For De Quervain’s tenosynovitis, the injection target is the distal first extensor compartment near the radial styloid. In this region, the SRN was found to traverse the first extensor compartment, transitioning from a volar position at Level C (−0.45 cm) to a dorsal position at Level D (+0.25 cm). This trajectory indicates that a needle approach from the dorsal aspect increases risk of encountering the SRN, whereas a volar-to-dorsal needle direction targeted at or distal to Level D (0.8 cm from the styloid process) may reduce the risk of nerve contact, as the nerve has migrated dorsally. This diagonal pattern aligns with an ultrasonographic study of Kim et al., who reported that the SRN crossed the first extensor compartment in a proximal–radial to distal–ulnar direction in 69% of De Quervain’s tenosynovitis patients [24]. Furthermore, Robson et al. reported in a cadaveric study that the first SRN branch arises at a mean of 4.92 cm proximal to the radial styloid, traveling directly over the first extensor compartment [13]. The volar needle approach is further supported by Lee et al., who demonstrated that SRN branches on the dorsal aspect are positioned closer to the first extensor compartment than those on the volar side [25].

Regarding sex differences, males showed significantly longer absolute longitudinal distances for *L*a, *L*b, and *L*c, which is primarily attributed to their longer total forearm length (26.6 ± 1.2 cm vs. 24.4 ± 1.0 cm, *p* < 0.001). However, the proportional ratios (*L*a/*L*, *L*b/*L*, *L*c/*L*, *L*d/*L*) did not differ significantly between sexes, indicating that the relative anatomical trajectory is consistent when normalized to forearm length. Furthermore, some depth measurements were significantly greater in men, likely reflecting sex-related differences in soft tissue and musculature thickness [26,27]. These findings suggest that while the same landmarks and proportional ratios can be universally utilized for both sexes, needle depth should be adjusted based on sex.

When implementing these injection approaches based on sonographic findings, clinicians should note that imaging representations may not always perfectly reflect the actual nerve microanatomy [28,29]. For instance, high-resolution ultrasound (HRUS) can aggregate smaller microscopic fascicles into a single visual unit, potentially obscuring exact histological boundaries. Therefore, while our proposed safe zones offer a reliable macro-anatomical framework, subtle discrepancies between ultrasound imaging and precise histology should be acknowledged with caution.

This study has several limitations. First, as detailed above, our safe injection corridors were established based on anatomical measurements in healthy volunteers, meaning these parameters may deviate under pathological conditions. A systematic review by Lee et al. reported a higher prevalence of internal septa dividing the compartment in De Quervain’s disease, which may severely constrict the fibro-osseous compartment and induce local tissue distortion [30]. Additionally, a prospective surgical study by Matzon et al. demonstrated variations in the number of tendon slips and tendon instability in De Quervain’s tenosynovitis [31]. Similarly, in intersection syndrome, Montechiarello et al. reported that peritendinous edema obliterates the normal hyperechoic tissue plane dividing the extensor compartments [32]. These structural alterations may shift the depth, tension, and exact course of the SRN. Therefore, while our study provides a valuable baseline, the direct application of these findings to clinical populations should be approached with caution.

Second, while intra-rater reliability was evaluated and demonstrated high reproducibility, an inter-rater reliability assessment could not be performed because all ultrasonographic examinations were conducted by a single examiner. Third, the relatively small sample size and recruitment from a single ethnic group may limit the generalizability of our findings. Further research involving larger and more diverse cohorts with inter-rater reliability evaluations is needed to validate and expand upon our results.

## 5. Conclusions

In this study, we identified the anatomical course of the SRN and defined a “safe zone” for wrist extensor compartment injections using high-resolution ultrasonography. For intersection syndrome, an injection corridor between 24.1% (Level A) and 12.7% (Level C) of the total forearm length proximal to the radial styloid process with a proximal-to-distal and dorsal-to-volar needle direction provides a relatively safe zone. For De Quervain’s tenosynovitis, a volar-to-dorsal needle direction targeted at or distal to 0.8 cm (Level D) from the radial styloid process may minimize the risk of nerve contact. Consequently, this differentiated, landmark-based approach may serve as a practical and customized clinical guide to mitigate iatrogenic nerve injury.

## Figures and Tables

**Table 1:** Baseline characteristics of the study participants.

Baseline Characteristics	Total (*N* = 29)
Sex, *N* (%)	
Male	15 (51.7%)
Female	14 (48.3%)
Age (years)	32.2 ± 6.7
Height (cm)	168.3 ± 6.9
Weight (kg)	63.5 ± 8.4
BMI (kg/m^2^)	22.4 ± 2.1
Total length of forearm (*L*) (cm) *	25.5 ± 1.5

Data are presented as Mean ± Standard deviation. * Total length of forearm is calculated based on 58 forearms (*N*: number of participants, BMI: body mass index).

**Table 2:** Anatomical measurements of the intersection area at Level A and B.

		Level A	Level B
Longitudinal distance from radial styloid process (*L*x) (cm)		6.2 ± 0.5	1.2 ± 0.4
Proportion of longitudinal distance to total forearm length (*L*x/*L*) (%)		24.1 ± 1.5	4.7 ± 1.6
Horizontal distance (cm)	Intersection point from radius (A1 or B1)	0.52 ± 0.13	0.32 ± 0.34
	SRN from radius (A2 or B2)	−0.71 [−0.84, −0.65] ^†^	0.08 [0.00, 0.17] ^†^
Depth (cm)	Intersection point	0.33 [0.29, 0.38] ^†^	0.32 [0.29, 0.36] ^†^
	SRN	0.50 ± 0.13	0.25 [0.21, 0.30] ^†^
	Superficial border of radius	0.41 ± 0.09	0.35 [0.32, 0.41] ^†^

Data are presented as Mean ± Standard Deviation or Median [Interquartile Range] ^†^. *L*x: Longitudinal distance from the radial styloid process, corresponding to *L*a (proximal intersection) and *L*b (distal intersection). Horizontal distance: Positive and negative values indicate that the structure is located on the dorsal or volar aspect of the superficial border of the radius, respectively. SRN: superficial radial nerve.

**Table 3:** Anatomical measurements of the crossing points of superficial radial nerve (SRN) over compartment I at Level C and D.

		Level C	Level D
Longitudinal distance from radial styloid process (*L*x) (cm)		3.2 ± 0.3	0.8 ± 0.4
Proportion of longitudinal distance to total forearm length (*L*x/*L*) (%)		12.7 ± 1.2	3.2 ± 1.8
Horizontal distance (cm)	Crossing point from radius (C1 or D1)	−0.45 ± 0.15	0.25 [0.14, 0.36] ^†^
Depth (cm)	Crossing point	0.33 ± 0.06	0.28 [0.24, 0.32] ^†^
	Superficial border of radius	0.48 [0.44, 0.54] ^†^	0.38 [0.34, 0.46] ^†^

Data are presented as Mean ± Standard Deviation or Median [Interquartile Range] ^†^. *L*x: Longitudinal distance from the radial styloid process, corresponding to *L*c (proximal SRN crossing) and *L*d (distal SRN crossing). Horizontal distance: Positive and negative values indicate that the structure is located on the dorsal or volar aspect of the superficial border of the radius, respectively.

**Table 4:** Generalized Estimating Equation (GEE) regression analysis assessing the linear relationships between total forearm length (*L*) and longitudinal distances of anatomical landmarks.

Variables	Predictor	Unstandardized Coefficient (*B*)	95% Wald Confidence Interval	*p*-Value
*L*a	*L*	0.205 **	[0.139, 0.272]	<0.001
*L*b	*L*	0.071 *	[0.012, 0.131]	0.019
*L*c	*L*	0.105 **	[0.048, 0.161]	<0.001
*L*d	*L*	−0.023	[−0.112, 0.066]	0.610

*L*a, *L*b, *L*c, and *L*d represent longitudinal distances from the radial styloid process at the defined levels. * *p* < 0.05, ** *p* < 0.001.

## Data Availability

The original contributions presented in this study are included in the article/supplementary material. Further inquiries can be directed to the corresponding author.

## Supplementary Materials

The following supporting information can be downloaded at: https://www.mdpi.com/article/10.3390/diagnostics16121788/s1, Table S1. Intraobserver reliability (ICC) of measurement parameters. File S1: Minimal dataset_revision.

## Abbreviations

The following abbreviations are used in this manuscript:

APL	Abductor pollicis longus
BR	Brachioradialis
ECRB	Extensor carpi radialis brevis
ECRL	Extensor carpi radialis longus
EPB	Extensor pollicis brevis
SRN	Superficial radial nerve
