# Supplementary material for: An Ultrasonographic Study of the Superficial Radial Nerve in Healthy Subjects: Suggesting a Safe Zone for Wrist Extensor Compartment Injections
Source: Diagnostics (Basel). 2026 Jun 10;16(12):1788. doi: 10.3390/diagnostics16121788 (PMC13298110; doi:10.3390/diagnostics16121788)
Supplement: Supplementary file 1 [file diagnostics-16-01788-s001.zip › Supplementary table_S1.pdf]

Supplementary Table S1. Intraobserver reliability (ICC) of measurement parameters.

Parameter	ICC	95% Confidence Interval
Longitudinal distance from radial styloid process		
La	0.918	[0.806 - 0.967]
Lb	0.894	[0.756 - 0.956]
Lc	0.921	[0.809 - 0.968]
Ld	0.921	[0.811 - 0.968]
Level A Measurements		
Horizontal distance of intersection point from radius (A1)	0.848	[0.658 - 0.937]
Horizontal distance of SRN from radius (A2)	0.917	[0.772 - 0.968]
Depth of intersection point	0.827	[0.618 - 0.927]
Depth of SRN	0.823	[0.606 - 0.926]
Depth of superficial border of radius	0.914	[0.684 - 0.970]
Level B Measurements		
Horizontal distance of intersection point from radius (B1)	0.886	[0.735 - 0.954]
Horizontal distance of SRN from radius (B2)	0.842	[0.621 - 0.936]
Depth of intersection point	0.755	[0.483 - 0.895]
Depth of SRN	0.843	[0.608 - 0.938]
Depth of superficial border of radius	0.777	[0.525 - 0.905]
Level C Measurements		
Horizontal distance of crossing point from radius (C1)	0.918	[0.799 - 0.967]
Depth of crossing point	0.817	[0.600 - 0.923]
Depth of superficial border of radius	0.906	[0.781 - 0.962]
Level D Measurements		
Horizontal distance of crossing point from radius (D1)	0.969	[0.924 - 0.988]
Depth of crossing point	0.879	[0.721 - 0.950]
Depth of superficial border of radius	0.877	[0.716 - 0.949]

La, Lb, Lc, and Ld represent longitudinal distances from the radial styloid process at the defined levels. SRN: superficial radial nerve, ICC: Intraclass Correlation Coefficient.
